# Hospitals and Water Supply

**Published:** 1911-11-25

**Authors:** 


*To the Editor of* The Hospital.

### HOSPITALS AND WATER SUPPLY.

*To the Editor of* The Hospital.

Sir,—The article that appeared in The Hospital of November 18, page 182, under the above heading, may be true so far as the provinces are concerned, but the statements contained therein are inaccurate if they are intended to apply to the metropolis. The Water Board cannot demand to charge a medical charity by measure. When the London Water Bill was under consideration in 1907 the Central Hospital Council approached the Government on the subject with the result that water is now supplied to hospitals at five per cent. on the rateable value. The gain to the hospitals of London will be seen by the following instance. Prior to the passing of the Act the Dreadnought Hospital paid an average of £190 per annum for water consumed at Greenwich, while the Albert Dock Hospital paid an average of £40. On the London Water Board being established the amount payable was reduced to a fixed sum of £30 at Greenwich, and £20 at the Albert Docks, a saving of £180 annually to the Seamen's Hospital Society. The hospitals of London have therefore to thank the Central Hospital Council for obtaining water for them at a cheap rate. The action of the Council must benefit the London hospitals by several thousands of pounds annually.

A London Hospital Officer.

